# The Effect of Therapeutic Exercise and Local Cryotherapy on Lower Limb Enthesitis in Non-Radiographic Axial Spondyloarthritis: A Case Report

**DOI:** 10.3390/jpm14101035

**Published:** 2024-09-28

**Authors:** Angelo Alito, Rossella Talotta, Valeria D’Amico, Daniela Amato, Demetrio Milardi, Paolo Capodaglio

**Affiliations:** 1 Department of Biomedical, Dental Sciences and Morphological and Functional Images, University of Messina, 98125 Messina, Italy; alitoa@unime.it (A.A.); dmilardi@unime.it (D.M.); 2 Rheumatology Unit, Department of Clinical and Experimental Medicine, University of Messina, 98125 Messina, Italy; rtalotta@unime.it (R.T.); dmcvlr95d43f158j@studenti.unime.it (V.D.); 3 Department of Physical and Rehabilitation Medicine, University Hospital “G. Martino”, 98125 Messina, Italy; daniela.amato@unime.it; 4 Research Laboratory in Biomechanics, Rehabilitation and Ergonomics, IRCCS, Istituto Auxologico Italiano, San Giuseppe Hospital, 28824 Verbania, Italy; 5 Department of Surgical Sciences, Physical Medicine and Rehabilitation, University of Torino, 10126 Torino, Italy

**Keywords:** enthesitis, exercise, local cryotherapy, non-radiographic axial SpA, rehabilitation

## Abstract

Background: Enthesitis is a common feature of spondyloarthritis and can severely impair the patient’s quality of life. International guidelines recommend multidisciplinary management of this condition, combining physical and pharmacological interventions. In this case report, we demonstrate clinical and ultrasonographic improvements by prescribing local cryotherapy and therapeutic exercise alone in an adult woman with non-radiographic axial SpA (nRX-AxSpA) complaining of heel enthesitis. Methodology: A personalized program was prescribed that focused on reducing pain, joint stiffness, and muscle tightness, improving strength and endurance. Pain, function, and degree of disability were assessed using the Numerical Rating Scale, the Victorian Institute of Sport Assessment-Achilles, the single-leg heel lift test, and the Foot Function Index. In addition, lower limb muscle strength was measured using a dynamometer and enthesitis was assessed ultrasonographically using the Glasgow Ultrasound Enthesitis Score System. Results: Benefits were evident as early as week 5 and persisted at 3 months on ultrasound assessment. No side effects were reported. Discussion: To the best of our knowledge, this is the first report of prescribing such a strategy in a patient with nRX-AxSpA. Given the good tolerability, this intervention could be considered in patients with contraindications to pharmacologic approaches.

## 1. Introduction

Spondyloarthritides (SpA) are a group of rheumatic diseases that share common pathogenic pathways, clinical manifestations, and therapeutic approaches. According to the classification criteria of the Assessment of SpondyloArthritis international Society (ASAS) [1,2], the predominant features of axial and peripheral SpA are inflammatory chronic back pain in patients under 45 years of age, evidence of sacroiliitis on imaging, presence of human leukocyte antigen (HLA)-B27 allele, enthesitis, dactylitis, peripheral synovitis, and various extra-articular conditions such as psoriasis, inflammatory bowel disease, uveitis, or previous infection. The HLA-B27 allele, together with infectious events, appears to play a crucial role in the pathogenesis of SpA by promoting mechanisms such as molecular mimicry, the unfolded protein response and autophagy, the activation of natural killer (NK) cells, CD4, and CD8 T lymphocytes, and the release of pro-inflammatory cytokines belonging to the interleukin (IL)-23/IL-17 axis [3,4]. In addition, repetitive mechanical stress at the entheses, which are the attachment points of tendons, ligaments, joint capsule, and fascia to bone [5], is considered a fundamental pathogenic factor in SpA [6].

Fibrocartilaginous entheses can be considered as organs that include bursae and fat pads in addition to the enthesis [6]. According to scientific evidence, the fibrocartilaginous entheses appear to be the first site affected by the inflammatory process in SpA. The most convincing hypothesis is that autoreactive cells such as macrophages, T-helper (Th)17, Th1 or Th22 lymphocytes infiltrate the fibrocartilaginous part of the axial or peripheral entheses, favoured by phenomena of neoangiogenesis, with the local release of pro-inflammatory mediators that may contribute to osteitis (e.g., IL-17), or entheseal calcifications (e.g., IL-22) [4,7]. The very late stage of SpA is indeed characterised by bone deposits at the entheseal sites with progressive limitation of the range of motion of the joint. This event is favoured by the activation of the Wingless-related integration site (Wnt) and bone morphogenic protein (BMP) signalling pathways, which promote the proliferation of mesenchymal cells and the differentiation of osteoblasts [4,7].

Given the complexity of the pathophysiological processes occurring in SpA, treatment requires a multidisciplinary approach combining pharmacological interventions with non-pharmacological methods such as education, physiotherapy, and psychological and emotional support [8,9].

From a pharmacological perspective, SpA patients can be treated with conventional, biological, or synthetic disease-modifying antirheumatic drugs (DMARDs), in addition to non-steroidal anti-inflammatory drugs (NSAIDS) or local glucocorticoid injections [10,11,12,13].

The treatment of enthesitis in psoriatic arthritis (PsA), which is part of the spectrum of SpA, has been specifically addressed in international guidelines. Overall, the latest recommendations from the American College of Rheumatology (ACR), the Group for Research and Assessment of Psoriasis and Psoriatic Arthritis (GRAPPA), and the European League Against Rheumatism (EULAR) include the use of NSAIDs, local glucocorticoid injections, methotrexate, or apremilast in treatment-naïve patients and bDMARDs targeting tumour necrosis factor-alpha (TNFα), IL-17, IL-12, and IL-23 or Janus kinase inhibitors (JAKi) as a further line of therapy in non-responders [10,11,14]. However, it is important to emphasise that these recommendations are based on a low to very low level of evidence. The 2021 GRAPPA recommendations also conditionally suggest physiotherapy for PsA enthesitis, although the evidence for efficacy from the studies available in the literature is limited [10].

Several studies also confirm the efficacy of bDMARDs targeting TNFα and IL-17, or JAKi in the treatment of enthesitis in patients with peripheral or axial SpA and ankylosing spondylitis (AS) [15,16,17,18].

Interestingly, bDMARDs targeting the IL-12/IL-23 axis were not effective in clinical trials in patients with AS enthesitis. This discrepancy may be related to a different cytokine milieu in axial and peripheral enthesitis, which could further complicate the choice of the best therapeutic approach for this SpA manifestation [19]. In addition, it should be emphasized that both NSAIDS, local glucocorticoid injections and conventional, biologic, or targeted synthetic DMARDs can lead to various adverse events, including opportunistic infections or malignancies. Long-term use of NSAIDs is particularly associated with gastrointestinal, renal, cardiovascular, and hepatic complications [20].

Corticosteroid injections can be burdened by local or systemic side effects, such as post-injection inflammatory reactions, infections, tendon ruptures, hypertension, osteoporosis and hyperglycemia [21].

The use of methotrexate frequently leads to gastrointestinal toxicity and, less frequently, to other complications, including malignancies [22].

Safety data from randomized controlled trials (RCTs) suggest that serious infections, malignancies, and cardiovascular events can rarely occur in SpA patients treated with anti-TNF and anti-IL-17 agents [23].

Observational studies also show that demyelinating diseases occur more frequently in SpA patients treated with anti-TNF compared to anti-IL-17 agents [23].

Finally, JAKi have been associated with an increased risk of herpes zoster virus reactivation, cardiovascular events, venous thromboembolism, and malignancy in cohorts with rheumatoid arthritis, but data on safety in the SpA population are limited [24].

On the other hand, exercise and physiotherapy are cornerstones of SpA treatment with a high to moderate level of evidence confirmed by new studies and an acceptable safety profile [24].

Non-pharmacological interventions are indeed more attractive for patients with comorbidities, poor compliance or contraindications to pharmacological therapies, and impaired function and quality of life.

In this context, physical therapy and rehabilitation could provide additional benefits by reducing disease activity and improving function. Physiotherapy and exercise can improve mobility, strength, and posture, and modalities such as laser therapy, ultrasound (US), or heat/cold can reduce pain and inflammation [25].

Cryotherapy, by reducing leukocyte and granulocyte tissue recruitment and macrophage infiltration following soft tissue injury, appears to decrease the metabolism, inflammation, and tissue damage that characterize the most common tendinopathies [26,27]. Evidence suggests that the ability of cold therapy to reduce pain in acute tendon injury may be due to its ability to reduce levels of prostaglandin E2 production in tendons, a highly active inflammatory molecule that causes pain and induces vasodilation and hyperalgesia [28].

However, the evidence for the use of cryotherapy is relatively weak and controversial, there is significant heterogeneity between studies, making them difficult to compare, and the number of RCTs remains small [29,30,31,32]. Although applying cold to the surface of the injured area may be effective in reducing pain or swelling, it could prolong the healing process because the human body sends signals to our inflammatory cells (macrophages) to release the hormone insulin-like growth factor (IGF-1), reducing blood flow and preventing the transport of these inflammatory chemicals and cells to the site of injury [33,34,35,36].

There is some evidence of the beneficial effect of local cryotherapy as an adjunctive treatment in inflammatory rheumatic diseases for symptom management, allowing better compliance with rehabilitation treatment, with a low-cost profile, simple administration, good tolerability, and safety profile [37]. As already mentioned, this approach could be used in patients with contraindications to systemic medications, particularly in long-term disease management, to avoid the side effects of chronic medication use [37].

Given the effects of cryotherapy on the proinflammatory cytokine cascade, it can be considered as part of a comprehensive management strategy for rheumatic diseases to relieve symptoms and improve function and quality of life [38,39].

In SpA-related enthesitis, the application of cryotherapy to the peripheral inflamed entheses could counteract the process of neoangiogenesis, which is the starting point for the recruitment of autoreactive cells. As a result, cryotherapy could prevent the upstream release of cytokines involved in inflammation and osteogenesis. The impairment of reparative processes culminating in calcification of the enthesis could therefore represent an additional benefit rather than an undesirable effect of such treatment. However, rehabilitation programs that focus specifically on SpA peripheral enthesitis are insufficiently described in the current literature. The authors report the case of a woman with non-radiographic axial (nr-ax) SpA and lower limb enthesitis who was effectively treated with physical exercise and local cryotherapy.

This case report followed the CARE guidelines for case reports [40].

## 2. Detailed Case Description

A 46-year-old Caucasian woman presented to our clinic in 2018 complaining of polyarticular pain and stiffness. Laboratory tests showed erythrocyte sedimentation rate (ESR) and C-reactive protein (CRP) levels above the upper limit and magnetic resonance imaging (MRI) of the hands and wrists revealed erosive arthritis and tenosynovitis of the extensor and flexor tendons. The patient also suffered from obesity, Hashimoto’s thyroiditis, type 2 diabetes mellitus, recurrent episodes of thrombophlebitis, iron deficiency, and obstructive sleep apnoea. As the predominant symptoms were allodynia and generalized hyperalgesia, with no detection of swollen joints or dactylitis on clinical examination, fibromyalgia was initially diagnosed and the patient was treated pharmacologically with nutritional supplements, NSAIDs, pregabalin, anxiolytics, and analgesics from 2018 to 2023. In February 2023, during a routine visit, we noted severe pain on palpation of the heel entheses and sacroiliac joints on both sides. At this time, the patient also complained of persistent morning stiffness, which improved during the day, as well as heel pain and stiffness when standing, such that she had to take several breaks and sit down.

Genotyping for HLA alleles, MRI of the sacroiliac joints, and X-rays of the ankles and feet were required to rule out the diagnosis of SpA. The results showed unilateral sacroiliitis on MRI, bilateral heel spurs on radiographs, and negativity for the HLA-B27 allele but positivity for the HLA-B35 and HLA-Cw6 alleles, which are associated with other forms of SpA [41]. Heel enthesitis was confirmed by US examination, which was performed in our clinic in April 2023. Indeed, US revealed a thickening of the calcaneal insertion of the left plantar fascia (4.9 mm) and calcification of the right Achilles tendon enthesis. No power Doppler signal or signs of bursitis were detected.

After a careful discussion of the therapeutic options, the woman agreed to be referred initially to a physiatrist to treat the enthesopathy in a nonpharmacological way. Hence, a personalized rehabilitation program was prescribed that focused on reducing pain, joint stiffness, and muscle tightness, improving strength, endurance, and joint range of motion (ROM), and normalizing the agonist/antagonist ratio of leg muscle strength to improve autonomy in walking and activities of daily living (ADLs).

Physiotherapy started at the end of April and consisted of 10 sessions, twice a week. The program started with passive and active movements of the tibio-tarsal joint and toes, active mobility exercises of the tibio-tarsal joint, and static stretching exercises of the lower limbs of both feet. From the 5th session onwards, strength exercises for the lower limbs, exercises to counteract resistance in plantar and dorsiflexion movements using elastic bands and exercises to re-adapt the load on the plantar fascia were added. At the end of each session, a localized cryo-compression program (program n°2) of 20 min on/off preset with light compression was applied to the left foot using a special ergonomic device (Cryotool, Fisiotools, Italy).

The decision to treat only the left foot with cryo-compression therapy was dictated by the US findings of thickening of the left plantar fascia and the patient’s subjective report of more pain on the left side than on the right.

The patient completed the entire physical program with optimal adherence to therapy and no adverse effects. At the end of the treatment, she reported an increase in pain-free standing time and a subjective improvement in lower limb muscle strength.

Functional and clinical assessments were performed at the beginning and at the end of the 5-week rehabilitation program. The following instruments were used: the Numerical Rating Scale (NRS) to measure pain [42]; the Victorian Institute of Sport Assessment-Achilles (VISA-A) to assess the severity of the condition and its impact on the patient’s life [43]; the single-leg heel lift test to assess plantar flexor strength and endurance [44]; and the Foot Function Index (FFI) to assess the impact of foot discomfort on the patient’s perceived health status in terms of pain, disability, and activity limitation [45]. In addition, a dynamometer (Biofet, Fisiotools, Italy) was used to objectively measure lower limb muscle strength and monitor progress over time. The test was performed three times for 5 s for each movement and the best result was measured in Newtons (N). The results and comparison between the two feet are shown in Table 1.

In addition, the patient underwent an US examination of the lower limbs’ entheses before the start of the physical intervention and after 3 and 6 months. The US examination was performed using a portable Esaote MyLab™Gamma US system with a linear probe frequency of 4 to 15 MHz.

The US findings (presence of bursitis, enthesophytes, altered echotexture and echogenicity, thickening of the enthesis, cortical bone erosion, and subcutaneous oedema) were scored according to the Glasgow Ultrasound Enthesitis Score System (GUESS) index developed by Balint et al. for the detection of lower limb enthesitis [46].

**Figure 1:**
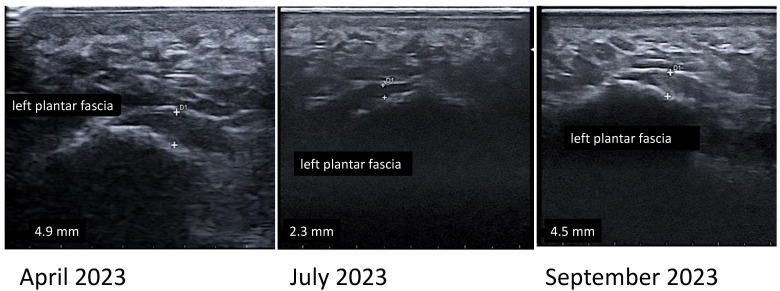
US assessment of left plantar fascia enthesis at baseline and 3 and 6 months after the start of the nonpharmacologic intervention.

At T0, the patient reached a total score of 2 (thickening of the left plantar fascia enthesis and calcification of the right Achilles tendon enthesis), which decreased to 1 in the following 3 months (Figure 1). This was due to the reduction in the thickness of the left plantar fascia attachment, while no changes were observed in the calcification of the right Achilles tendon enthesis. However, 6 months after the start of the intervention, the thickening of the left plantar fascia enthesis returned to values of 4.5 mm (GUESS score 2).

## 3. Discussion

Entheses play a crucial role in connecting soft tissues and bones by dissipating the force generated by muscle contraction and ensuring joint stability. They can be divided into fibrous and fibrocartilaginous entheses, depending on the tissue present at the bone attachment site [47]. Fibrocartilaginous entheses are more likely to be subjected to repetitive mechanical stress, which can cause enthesopathies. This type of enthesis is characterized by four zones (pure dense fibrous connective tissue, uncalcified fibrocartilage, calcified fibrocartilage, and bone) that follow one another in a continuous gradient [47]. Each of these four zones is characterized by a specific microenvironment in terms of the type of cells and the composition of the extracellular matrix. Type I collagen and proteoglycans, present in pure dense fibrous connective tissue/subchondral bone and uncalcified/calcified fibrocartilage, respectively, are the main players in ensuring adequate transmission of mechanical forces to the bone. Inflammatory conditions such as SpA, as well as other genetic or environmental factors, can alter the cellular and extracellular composition by increasing the degree of vascularization, promoting the recruitment of immune cells with local inflammation, and inducing the final process of proliferation and mineralization [48]. In SpA, favourable genetics, the deposition of microbial products on the entheses, biomechanical stress, adipokines, dysbiosis or gut hormones are considered risk factors for the development of enthesitis [4,7,49]. Interestingly, it appears that entheses have resident immune cells that include innate lymphoid cells type 3 (ILC3), g/d T lymphocytes, and various subsets of Th lymphocytes, which together may produce cytokines such as TNF- α, IL-17, IL-23, and IL-22 [50]. These cytokines play a physiological role in the process of tissue repair in degenerative tendinopathies, favouring the calcification of soft tissues. In SpA, the abnormal production of such mediators would favour a chronic inflammatory and ossification process.

Despite the central role that entheses play in the pathogenesis of SpA, the prevalence of enthesitis is reported to be 13.6–35%, with the Achilles tendon and plantar fascia being the most affected sites [51]. Studies show that enthesitis is significantly correlated with higher disease activity and worse functional outcomes in both axial and peripheral forms of SpA [51,52].

Heel enthesopathy can have an important functional impact on people with SpA. Inflammation of the Achilles tendon and plantar fascia entheses can cause pain, tenderness, and swelling in the affected area, impairing walking ability and thus leading to reduced mobility and disability [53]. The persistence of this problem can result in tendon damage and bone structural changes (e.g., enthesophytes) [54]. Therefore, prompt diagnosis and multidisciplinary treatment are indeed necessary to reverse those changes and promote healing [53].

The clinical diagnosis of enthesitis is often difficult as many other painful conditions can mimic this condition. In addition to the physical examination, which should indeed be the first step in the recognition and evaluation of enthesitis, musculoskeletal US can be another reliable tool for describing entheseal anatomical changes [55].

According to the Outcome Measures in Rheumatology (OMERACT), US examination of the entheses in SpA patients should focus on hypoechogenicity, increased thickness, bone erosions, calcifications or enthesophytes, and power Doppler signal [56]. Among the various US scoring systems for SpA enthesitis, the GUESS method has shown a sensitivity of 96.7% and a specificity of 80% and allows a good differentiation between enthesitis and mechanical enthesopathy [57]. Due to its high reliability in lower limb enthesitis, this method could be used in daily clinical practice to assess the presence of clinical or subclinical Achilles or plantar fascia enthesitis in SpA patients and to monitor response to therapies.

Treatment of SpA enthesopathy aims to promote healing and manage pain with pharmacologic (e.g., NSAIDs, systemic steroids or local steroid injections, biologic, or targeted DMARDs) and nonpharmacologic interventions, which may include physical therapy and therapeutic exercise, as well as other techniques such as cryotherapy.

In fact, cryotherapy could play an important role in the treatment of enthesitis in patients affected by SpA, which is characterized by inflammation that contributes to significant pain, stiffness, and functional impairment [58].

Cold therapy induces vasoconstriction, which constricts blood vessels and reduces blood flow to the inflamed area, leading to a reduction in the local production of inflammatory mediators, thereby limiting the inflammatory process, minimizing tissue damage, and promoting healing [59].

In addition, it may provide an analgesic effect by slowing nerve conduction velocity, thereby reducing the transmission of pain signals and significantly improving adherence to treatment [60].

Cold therapy can reduce muscle spasms and secondary muscle tension around the affected entheses, which often exacerbate pain and mobility problems, further contributing to pain relief and improved functional outcomes [61]. The combination of several interventions can improve strength, restore joint flexibility and mobility, correct biomechanical imbalances, and promote a gradual return to normal activity [62].

This case report demonstrates that the combination of two nonpharmacologic interventions consisting of therapeutic exercise and local cryotherapy can provide mid-term clinical and structural benefits in Achilles and plantar fascia SpA enthesopathy. In addition, the procedure was well tolerated and no adverse events were recorded.

To the best of our knowledge, this is the first report of prescribing such a strategy for a patient with nRX-AxSpA. The benefits of local cryotherapy have been described in cases of nonSpA epicondylitis and patellar ligament enthesopathy [63,64]. A paper by Guillot et al. also reported a reduction in synovial levels of IL-6, IL-1, and vascular endothelial growth factor (VEGF) in patients with SpA after local ice cryotherapy sessions [38].

Vasoconstriction and the change in the amount of pro-inflammatory mediators could be the basis for the improvement in structural US changes at 3 months and the reduced degree of pain perception at 5 weeks observed in our study.

However, some limitations must be considered. Tendons present a complex healing process that involves structural, cellular, and vascular changes over time [65,66]. During the follow-up, the natural tendon healing process, which implies a gradual decrease in inflammatory and pain mediators, could also account for the reduction in pain and function. The effect of ice or US could be negligible compared with the time factor. The authors indeed observed clinical and structural improvements occurring as early as weeks 5 and 12, respectively, but the structural effects seemed to be lost in the long term (6 months). These data could highlight the importance of maintaining rehabilitation treatment until symptoms resolve, followed by a personalized physical activity and exercise program to maintain the improvements achieved. Secondly, although the patient experienced clinical improvement, we could not demonstrate a structural benefit of therapeutic exercise alone on calcification, on which the effects of local cryotherapy were not tested. Finally, the effects of our approach on the synovio-entheseal complex were not studied.

## 4. Conclusions

This case report highlights the importance of physical rehabilitation in SpA patients. The use of cryotherapy in addition to therapeutic exercise may provide an alternative to local or systemic medication in the treatment of peripheral enthesitis, provided that the treatment is carried out over time. This approach could be integrated into early-stage management, post-exercise, recurrent pain, acute inflammatory episodes or as part of a multimodal treatment plan that includes rest, medication, and physiotherapy, allowing treatment to be personalized after ongoing assessment to individualize treatment protocols, also based on patient response. Patients who are best suited to cryotherapy include those who respond poorly to other pharmacological strategies, those who do not consent to such treatment, or those for whom there is a contraindication. Local cryotherapy has minimal costs associated with ice packs or dedicated cryotherapy devices, which are relatively inexpensive, easily reusable, and widely available. This cost-effectiveness, combined with its clinical efficacy and safety profile, makes cryotherapy a valuable adjunctive treatment in the management of enthesitis, potentially reducing the overall financial burden on both patients and healthcare systems by reducing the need for more costly interventions (e.g., analgesics) and frequent medical consultations. It also appears useful to tailor cold therapy to the stage of the tendon healing process and the pain experienced by the patient.

## Figures and Tables

**Table 1:** Functional and clinical scores before and after the rehabilitation treatment.

	Before Treatment		After 5-Week Treatment	
	Left Foot	Right Foot	Left Foot	Right Foot
NRS (0-10)	9	7	0	4
VISA-A (100-0)	16.5%	27.5%	70%	56%
Single-leg heel rise test (reps max/time)	6/64 s	4/11.5 s	15/28 s	12/22 s
FFI (0-100)	90.50%	87.60%	21.7%	39.4%
Dynamometer				
Plantar flexion (N)	10.8	9.4	11.6	12.5
Dorsal flexion (N)	6.4	10.9	12.9	8.4

FFI, Foot Function Index; N, Newton; NRS, Numerical Rating Scale; VISA-A, Victorian Institute of Sport Assessment-Achilles.

## Data Availability

The data presented in this study are available within the article.
